# Transgenic Model Systems Have Revolutionized the Study of Disease

**DOI:** 10.1089/dna.2021.0514

**Published:** 2022-01-12

**Authors:** Alexandre Mayran, Christopher Chase Bolt

**Affiliations:** School of Life Sciences, Ecole Polytechnique Fédérale de Lausanne (EPFL), Lausanne, Switzerland.

**Keywords:** transgenesis, genetic modification, biotechnology, model organism, COVID-19

## Abstract

The current pandemic caused by severe acute respiratory syndrome coronavirus 2 (SARS-CoV-2) has affected most of the world in a profound way. As an indirect consequence, the general public has been put into direct contact with the research process, almost in real time. Justifiably, a lot of this focus has been targeted toward research directly linked to coronavirus disease 2019 (COVID-19). In this opinion article, we want to highlight to a general audience the value of having a diverse “portfolio” of research approaches for society as a whole. In this study, we will focus on our field of research, namely the study of gene regulation through the use of transgenesis. We will highlight how this type of research can also be used to provide a better understanding as well as tools to fight SARS-CoV-2 and other future challenges.


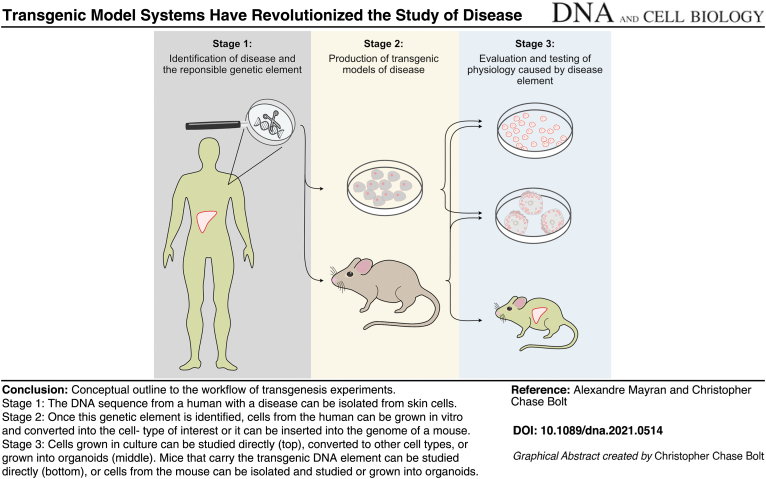


## Introduction

Transgenesis is, in the broadest sense, a set of techniques used to transfer a piece of genetic material from the genome of one species into the genome of another. The transferred genetic material is generally a combination of a gene and small DNA fragment that precisely controls how or when the transgene is expressed. Some examples of transgenesis have become familiar to the general public as commercial products found in the produce section of the grocery store. These are the commonplace fruits and vegetables known as genetically modified organisms. In many of these examples, the fruits and vegetables are transgenic for genes that make them more shelf-stable or disease-resistant products (Lobato-Gómez *et al.*, 2021).

But in biomedical research, transgenesis is a tool essential for learning how DNA and the genome are involved in normal and pathological conditions. One common application of transgenesis is to “humanize” animal models to study human gene (or genome) function by reproducing certain human pathologies in a research model organism (Justice *et al.*, 2011). This powerful combination of genetics and biotechnology allows researchers to perform valuable experiments in mice such as performing screens for novel compounds useful in fighting disease, test hypotheses that would otherwise be impossible, and to understand the molecular origins of many human diseases.

The humble house mouse has proven to be an extraordinarily valuable animal model for a variety of human diseases ranging from anorexia to obesity, and from development to aging. In previous decades, genetic research in mice was limited to the study of human diseases to which mice were also susceptible, either by nature or through a lengthy and expensive process of random mutagenesis experiments, often with no certainty that valuable insights would be made (Culiat *et al.*, 1997). With the discovery of transgenesis technologies (Palmiter *et al.*, 1982; Palmiter and Brinster, 1985), it became possible to directly produce mice with human pathologies, and so over the following decades, this became a predominant way to learn about normal and abnormal physiology (Doyle *et al.*, 2012).

More recently, technological developments and gained knowledge have made it possible to produce organoids, organ-like tissues grown *in vitro* directly from a small sample of cells taken from a patient. This nascent world of organoid research has expanded the toolbox of methods used to study the relationship between genes and disease, simultaneously providing researchers with a model for organ development and a way to measure the impact of genetic alterations. The identification of DNA elements relevant to a particular physiology remains one of the most challenging aspects of modern genomics, but with new technologies continuously arising and combined with transgenesis techniques, continuous progress is being made.

### Making a transgene

#### Identification and testing of genetic elements

In 2001, the international consortium of the Human Genome Project completed the first-draft sequencing of the human genome (Lander *et al.*, 2001). Before its completion it was widely believed that having a map of the human genome would provide clear explanations for many human genetic disorders. Instead, it showed that protein-coding genes only account for about 1–2% of the whole genome, whereas the remainder of the DNA sequence appeared devoid of information and was sometimes colloquially referred to as “junk DNA,” which is where the vast majority of spontaneous DNA mutations occur.

Since those early days, we have come to understand that these regions are not junk, but contain a dense variety of DNA elements that directly control or interfere with how the genes are expressed (ENCODE Project Consortium, 2012). In time, new methodologies arose that allowed for association of the mutations with diseases, but mechanistic relationships between these two could not be assigned based on these methods alone (Auton *et al.*, 2015; Zhang and Lupski, 2015).

The falling cost of next-generation high-throughput sequencing has led to new large-scale efforts dedicated to the identification of functional noncoding elements through biochemical approaches or to the association of individual genetic variation with tissue-specific gene expression (Lonsdale *et al.*, 2013). These consortiums have generated large databases of potentially functional elements. At the other end of this genome-wide scale, other experimental approaches are highly specific and evaluate the function of specific genomic elements.

These range from assessing the activity of specific regulatory elements in mouse embryos through large-scale transgenesis experiments (Visel *et al.*, 2007), to generating transgenic mice that carry genetic alterations that reproduce human diseases. With the advent of CRISPR-based technologies these approaches have become tenable in recent years allowing for the precise modeling of human mutations in mice allowing researchers to evaluate the specific molecular events that occur during the formation of disease (Kraft *et al.*, 2015). Regardless of the specific approach taken by a particular research group, all of these approaches complement each other to produce a growing body of knowledge that helps us understand the relationship between genetic mutation and disease.

#### Generation of transgenics

Once a relevant genetic element has been identified and suspected to be causative of a particular condition it can be tested using the process of transgenesis. The applications of transgenesis vary a lot, but the central goal of these types of experiments is to evaluate the functional importance of a particular genetic element in a physiological context such as an infection or developmental process. This genetic transfer can be done to study processes *in vitro*, in which case cells grown in flasks will be used as recipients for the transferred DNA. This is both rapid and amenable to high-throughput testing, to measure how the element responds in cells that are also treated with drugs or other small molecules.

In this case, the introduction of a foreign DNA element can be mediated by putting the cells in a solution containing the DNA fragment and then applying an electrical current. This causes the DNA to enter the cells where it will integrate into genomic DNA. Alternatively, the DNA can be encapsulated within microscopic bubbles made of synthetic lipids that can fuse with cellular membranes to deposit the DNA into the cellular cytoplasm (Hou *et al.*, 2021). Also, this can be performed *in vivo*; the process to generate a living mouse that carries a transgene involves the injection of transgene elements directly into the fertilized egg.

With all of these techniques, transgenic DNA that enters the cell tends to insert into the genomic DNA at random locations. This can be problematic because much of the genome has some biochemical activity that can interact with the transgene making results difficult to interpret precisely.

One way around this problem is to specify where the transgenic DNA should be inserted. This is achieved by using components of the CRISPR-Cas9 system along with the transgene. In this context CRISPR-Cas9 is targeted to a particular region of the genome where it will cut the genomic DNA. When the genomic DNA is broken like this, it increases the likelihood that the transgenic DNA will insert at the targeted location (Li *et al.*, 2020). This advancement makes it possible to substitute DNA elements such as genes, to repair damaged DNA, and to create tools to study how genes behave.

The types of transgenic DNA elements and the methods used to produce them vary depending on the question of the researcher. A recent timely example involves the generation of a transgenic mouse model to study coronavirus disease 2019 (COVID-19) (Sun *et al.*, 2020). Indeed, the severe acute respiratory syndrome coronavirus 2 (SARS-CoV-2) virus does not normally infect mice well because the Spike protein of the virus is not compatible with the mouse ACE2 receptor, which is the protein allowing entry of the SARS-CoV-2 virus into a cell (Dinnon *et al.*, 2020). By inserting the human ACE2 gene into the mouse genome mice became susceptible for SARS-CoV-2, thus providing an invaluable tool to study COVID-19.

## Future Perspectives

There is a sincere and rational hope that scientists soon will be able to grow complex tissues *in vitro* to replace damaged ones within our bodies. Indeed, much of the work is still in the earliest stages, such as *in vitro* grown organoids, but rapid advances are being made (Kim *et al.*, 2020). Whatever applications derive from these methods, much of the foundational knowledge enabling their development came from simple transgenesis experiments, such as one to engineer human skin cells into stem cells (Takahashi and Yamanaka, 2006). It is remarkable that much of this work has been done within the past 15 years.

In addition, these methods also hold strong potential to improve personalized medicine. Reproducing individual mutations or generating organoids from patient cells could provide a patient-specific tool to test drugs or even screen them before injecting them into the patient, allowing tailored treatment adapted to the patient's unique genetic context. One specific application concerns a genetic risk factor to severe COVID-19. A genomic segment inherited from Neanderthals was found to be the major driver of severe COVID-19 (Zeberg and Pääbo, 2020).

Organoid derivation from patients harboring this mutation, or engineering this genetic segment into existing models of cells capable of producing lung organoids could help researchers better understand what drives this increased risk of severe COVID-19. The transgenic approach would allow a much better controlled experimental framework as the only genetic difference between cells not at risk and those at higher risk would be restricted to this Neanderthal inherited genetic segment.

These benefits also extend far beyond human medicine. Already much of the world has benefitted from transgenic plants that increase the density of food crops, and allow growth of disease and drought resistance species. These developments, and their continued improvements will be important to stabilizing the production agricultural products in a future where climatic and population changes are likely to be profound. Yet these technologies hold even more potential; for example, there may be a future where the use of transgenes can restore some of the ecosystems devastated by climate change or even to bring back extinct species (Seddon *et al.*, 2014; Shapiro, 2015).

## Glossary

Gene: Often described as a hereditary segment of DNA, a portion of chromosome that encodes for protein sequences.

Genome: The complete set of DNA sequences of an organism.

Gene regulation: Mechanisms that cells use to control the expression of genes.

Mutations: Alteration of DNA sequences, can be detrimental, neutral, or beneficial for the organism.

Humanization: The process of genetically altering a nonhuman species to reproduce processes normally that would only occur in human.

*In vitro*: As opposed to *in vivo*, refers to research that is performed without the use of live organisms, for example, using cell culture or organoids, as well as the study of biochemical reactions in a laboratory, outside of cells.

Organoid: Refers to a mass of cells grown *in vitro* as a complex 3D structure that mimics the organization and function of an organ.

CRISPR/Cas9: It is an adaptive immune response system that bacteria evolved to acquire protection against viral infection. In recent years, it was adapted to become a versatile toolbox for easy genome editing.

Transgene: Refers to the insertion of a foreign piece of DNA into the genome of an organism.

## References

[B1] Auton, A., Abecasis, G.R., Altshuler, D.M., Durbin, R.M., Abecasis, G.R., Bentley, D.R., *et al.* (2015). A global reference for human genetic variation. Nature 526**,** 68–74. 26432245 10.1038/nature15393 PMC4750478

[B2] Culiat, C.T., Carver, E.A., Walkowicz, M., Rinchik, E.M., Cacheiro, N.L.A., Russell, L.B., *et al.* (1997). Induced mouse chromosomal rearrangements as tools for identifying critical developmental genes and pathways. Reprod Toxicol 11**,** 345–351. 9100310 10.1016/s0890-6238(96)00147-5

[B3] Dinnon, K.H., Leist, S.R., Schäfer, A., Edwards, C.E., Martinez, D.R., Montgomery, *et al.* (2020). A mouse-adapted model of SARS-CoV-2 to test COVID-19 countermeasures. Nature 586**,** 560–566. 32854108 10.1038/s41586-020-2708-8 PMC8034761

[B4] Doyle, A., McGarry, M.P., Lee, N.A., and Lee, J.J. (2012). The construction of transgenic and gene knockout/knockin mouse models of human disease. Transgenic Res 21**,** 327–349. 21800101 10.1007/s11248-011-9537-3 PMC3516403

[B5] ENCODE Project Consortium. (2012). An integrated encyclopedia of DNA elements in the human genome. Nature 489**,** 57–74. 22955616 10.1038/nature11247 PMC3439153

[B6] Hou, X., Zaks, T., Langer, R., and Dong, Y. (2021). Lipid nanoparticles for mRNA delivery. Nat Rev Mater 1–17. DOI: 10.1038/s41578-021-00358-0. PMC8353930 34394960

[B7] Justice, M.J., Siracusa, L.D., and Stewart, A.F. (2011). Technical approaches for mouse models of human disease. Dis Model Mech 4**,** 305–310. 21558063 10.1242/dmm.000901 PMC3097452

[B8] Kim, J., Koo, B.-K., and Knoblich, J.A. (2020). Human organoids: model systems for human biology and medicine. Nat Rev Mol Cell Bio 21**,** 571–584. 32636524 10.1038/s41580-020-0259-3 PMC7339799

[B9] Kraft, K., Geuer, S., Will, A.J., Chan, W.-L., Paliou, C., Borschiwer, M., *et al.* (2015). Deletions, inversions, duplications: engineering of structural variants using CRISPR/Cas in mice. Cell Rep 10**,** 833–839. 25660031 10.1016/j.celrep.2015.01.016

[B10] Lander, E.S., Linton, L.M., Birren, B., Nusbaum, C., Zody, M.C., Baldwin, J., *et al.* (2001). Initial sequencing and analysis of the human genome. Nature 409**,** 860–921. 11237011 10.1038/35057062

[B11] Li, H., Yang, Y., Hong, W., Huang, M., Wu, M., and Zhao, X., (2020). Applications of genome editing technology in the targeted therapy of human diseases: mechanisms, advances and prospects. Signal Transduct Target Ther **5,** 1. 10.1038/s41392-019-0089-y PMC6946647 32296011

[B12] Lobato-Gómez, M., Hewitt, S., Capell, T., Christou, P., and Dhingra, A. (2021). Transgenic and genome-edited fruits: background, constraints, benefits, and commercial opportunities. Hortic Res **8,** 166. 10.1038/s41438-021-00601-3 PMC8286259 34274949

[B13] Lonsdale, J., Thomas, J., Salvatore, M., Phillips, R., Lo, E., Shad, S., *et al.* (2013). The genotype-tissue expression (GTEx) project. Nat Genet 45**,** 580–585. 23715323 10.1038/ng.2653 PMC4010069

[B14] Palmiter, R.D., and Brinster, R.L. (1985). Transgenic mice. Cell 41**,** 343–345. 2985274 10.1016/s0092-8674(85)80004-0

[B15] Palmiter, R.D., Brinster, R.L., Hammer, R.E., Trumbauer, M.E., Rosenfeld, M.G., Birnberg, N.C., *et al.* (1982). Dramatic growth of mice that develop from eggs microinjected with metallothionein–growth hormone fusion genes. Nature 300**,** 611–615. 6958982 10.1038/300611a0 PMC4881848

[B16] Seddon, P.J., Moehrenschlager, A., and Ewen, J. (2014). Reintroducing resurrected species: selecting DeExtinction candidates. Trends Ecol Evol 29**,** 140–147. 24513302 10.1016/j.tree.2014.01.007

[B17] Shapiro, B. (2015). Mammoth 2.0: will genome engineering resurrect extinct species? Genome Biol **16,** 228. 10.1186/s13059-015-0800-4 PMC4632474 26530525

[B18] Sun, S.-H., Chen, Q., Gu, H.-J., Yang, G., Wang, Y.-X., Huang, X.-Y., *et al.* (2020). A mouse model of SARS-CoV-2 infection and pathogenesis. Cell Host Microbe 28**,** 124–133.e4. 32485164 10.1016/j.chom.2020.05.020 PMC7250783

[B19] Takahashi, K., and Yamanaka, S. (2006). Induction of pluripotent stem cells from mouse embryonic and adult fibroblast cultures by defined factors. Cell 126**,** 663–676. 16904174 10.1016/j.cell.2006.07.024

[B20] Visel, A., Minovitsky, S., Dubchak, I., and Pennacchio, L.A. (2007). VISTA Enhancer Browser—a database of tissue-specific human enhancers. Nucleic Acids Res 35**,** D88–D92. 17130149 10.1093/nar/gkl822 PMC1716724

[B21] Zeberg, H., and Pääbo, S. (2020). The major genetic risk factor for severe COVID-19 is inherited from Neanderthals. Nature 587**,** 610–612. 32998156 10.1038/s41586-020-2818-3

[B22] Zhang, F., and Lupski, J.R. (2015). Non-coding genetic variants in human disease. Hum Mol Genet 24**,** R102–R110. 26152199 10.1093/hmg/ddv259 PMC4572001

